# A Case of Cryptorchidism with Ipsilateral Congenital Unilateral Absence of the Vas Deferens and Contralateral Renal Agenesis

**DOI:** 10.1155/2016/2379793

**Published:** 2016-08-11

**Authors:** Young Dong Yu, Young Kwon Hong

**Affiliations:** Department of Urology, CHA Bundang Medical Center, CHA University, Seongnam 13497, Republic of Korea

## Abstract

*Introduction and Aims*. Congenital absence of the vas deferens is an uncommon anomaly and this clinical condition is responsible for up to 1-2% of male infertility. It can be either unilateral or bilateral and the associated anomalies include cryptorchidism, seminal vesicles and ejaculatory ducts anomalies, and renal anomalies such as renal agenesis. We hereby present a case of congenital unilateral absence of vas deferens, which was found incidentally during an evaluation of undescended testis in a patient with ipsilateral renal agenesis.* Case Presentation*. A 10-month-old boy was referred to the urology clinic with an undescended right testis. Preoperative abdominal ultrasonography showed agenesis of the right kidney and the absence of right vas deferens and epididymis was confirmed during laparoscopic orchiectomy performed due to short right spermatic cord. There were no other concomitant anomalies of the genitourinary system observed in evaluation.* Conclusion*. Congenital unilateral absence of the vas deferens with cryptorchidism and renal agenesis is a rare diagnostic entity. Cryptorchidism or absent vas deferens found incidentally should lead the physician to evaluate the status of the contralateral vas deferens and conduct a renal tract ultrasound study.

## 1. Introduction

Congenital unilateral absence of the vas deferens (CUAVD) is an uncommon anomaly, which may contribute to male infertility and it has been associated with renal agenesis and a variety of other anomalies that was first described in 1870 by Reverdin [1]. The anomalies associated with CUAVD include seminal vesicles and ejaculatory ducts anomalies, cryptorchidism, malrotation of the solitary kidney, multicystic kidney, ectopic kidney, and horseshoe kidney and they are often asymptomatic so that they are diagnosed incidentally during orchidopexy or surgical exploration for inguinal hernias or even during evaluation for infertility in adults. A reported prevalence range of CUAVD in male population is 0.5%–1% [2, 3]. Therefore, it is important to be aware of this condition to uncover coexisting anomalies in the patients with absence of the vas deferens or undescended testis. Here, we present a CUAVD case, which was found incidentally during an evaluation of undescended testis in a patient with ipsilateral renal agenesis.

## 2. Case Presentation

**Figure 1:**
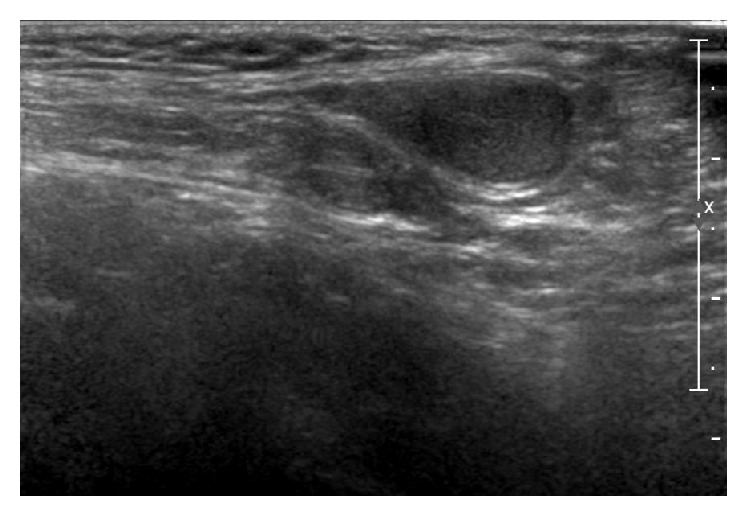
Left epididymis and testis with no pathological signs seen in scrotal ultrasonography.

**Figure 2:**
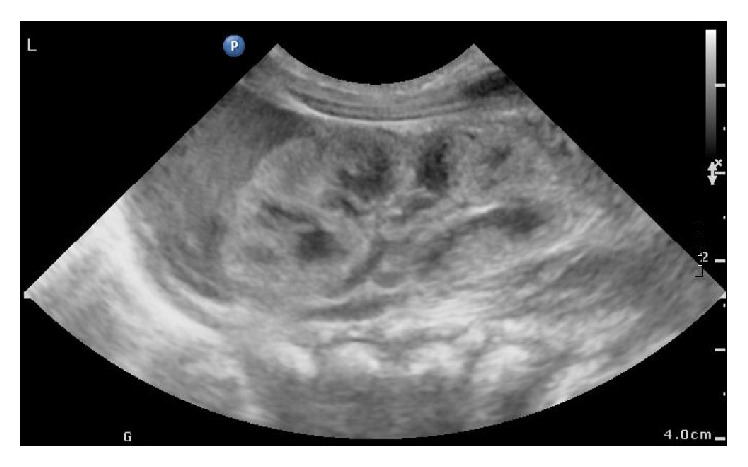
Normal looking left kidney in abdominal ultrasonography.

**Figure 3:**
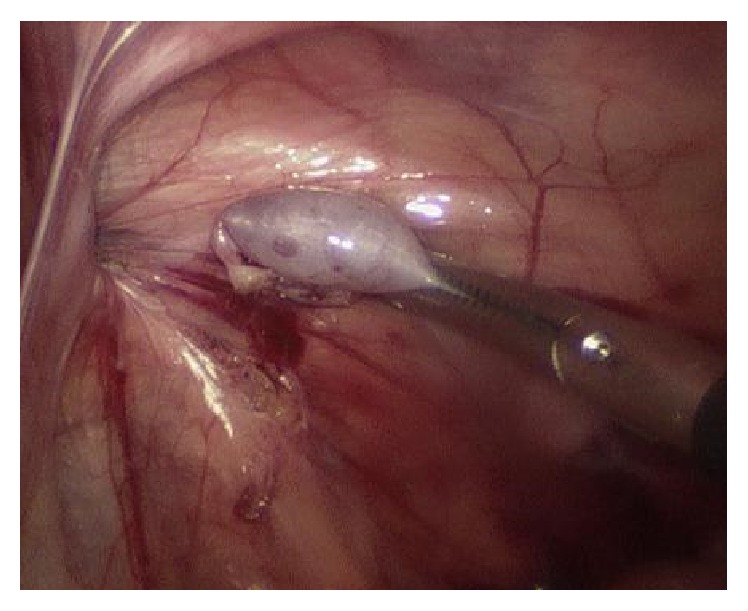
Absence of right vas deferens and epididymis in laparoscopic surgery.

A 10-month-old boy was referred to the urology clinic with an undescended right testis. A presurgical history of the patient revealed that the patient had agenesis of the right kidney and no other concomitant anomalies of the genitourinary system had been evaluated before surgery. The clinical examination presented orthotopically positioned left testis but right testis was palpable neither in the inguinal region nor in scrotum. Left hemiscrotum showed no redness or swelling and left epididymis was also verified by palpation. The inguinal area had no palpable mass referring to inguinal hernia. However, an absence of the vas deferens on the right hemiscrotum was found. Then a scrotal ultrasonography was undertaken and it confirmed cryptorchidism of right testis located at the level of right internal inguinal ring and the suspected diagnosis of agenesis of the right epididymis. Left epididymis and testis had no pathological signs seen (Figure 1). Moreover, abdominal ultrasonography revealed that renal agenesis on the right side and left kidney accompanied no compensatory hypertrophy or hydronephrosis (Figure 2). Laboratory test presented creatinine level of 0.75 mg/dL proving normal kidney function. After informed consent was obtained from the patient's parents, the patient underwent a laparoscopic surgery for right orchiopexy and absence of right vas deferens and epididymis was noted (Figure 3). However, due to relatively short length of right spermatic cord structure, laparoscopic orchiectomy was performed instead of orchiopexy. The patient's postoperative histologic analysis of right testis confirmed a normal testicle with absent epididymis.

## 3. Discussion

Development of renal system is closely integrated with the development of genital system. The ureteric bud develops from the mesonephric duct during the 5th week of gestation and the elongated stalk of the ureteric bud that is called the metanephric duct later forms the ureter. Moreover, the ingrowth of the branching ureteric buds into the metanephric blastema results in the characteristic lobulated appearance of the definitive kidney. The mesonephric duct differentiates into the bladder trigone, the seminal vesicle, the ductus deferens, and the distal two-thirds of the epididymis. An essential requirement for renal genesis after induction and differentiation of the intermediate mesoderm is the development of the ureteric bud at the caudal area of the mesonephric duct. The possible reasons for renal agenesis include the lack of induction of the metanephric blastema by the ureteral bud, primary absence of the caudal nephrogenic core, ureteral bud malformation, and dysplasia of mesonephric duct [4–6]. Congenital absence of the vas deferens (CAVD) can be either unilateral (CUAVD) or bilateral (CBAVD) and this clinical condition is responsible for up to 1-2% of male infertility. Most of these male infertility cases are due to bilateral vas agenesis (1%–6%) and only 0.4% of male infertility cases have been due to CUAVD. CAVD is considered a primary genital form of cystic fibrosis and up to 80% of patients with CAVD have mutations associated with Cystic Fibrosis Transmembrane Conductance (CFTR) gene [7]. In addition, Casals et al. [8] represented that 38% of CAVD cases are related to CFTR gene mutation. However, Schlegel et al. [9] reported that a CFTR gene abnormality in CAVD patients with renal agenesis occurs very rarely. Thus, in patients with cryptorchidism, the presence of vas deferens should be evaluated during scrotal palpation and abdominal ultrasound study is also recommended to evaluate any other coexisting abnormalities such as renal agenesis. In addition, CFTR gene analysis might be also helpful to evaluate the presence of latent cystic fibrosis. For adult patients with CAVD, vasography or transrectal ultrasonography can also be utilized to visualize a missing segment of vas deferens and fertility exam is also strongly recommended.

## 4. Conclusion

CUAVD with cryptorchidism and renal agenesis is a rare diagnostic entity. The exact pathophysiology of CUAVD still remains poorly understood. The absent vas deferens found in scrotal or laparoscopic abdominal surgery should lead the surgeon to explore the status of the contralateral vas deferens and conduct a renal tract ultrasound study. Genetic study for the evaluation of CFTR gene might be also helpful to evaluate the presence of latent cystic fibrosis. Moreover, scrotal palpation to confirm the presence of vas deferens should be recommended as a part of the routine physical exam in males.

## References

[1] Schukfeh N., Kuebler J. F., Schirg E. (2008). Dysplastic kidney and not renal agenesis is the commonly associated anomaly in infants with seminal vesicle cyst. *BJU International*.

[2] Weiske W.-H., Sälzler N., Schroeder-Printzen I., Weidner W. (2000). Clinical findings in congenital absence of the vasa deferentia. *Andrologia*.

[3] Khan Z. A. J., Novell J. R. (2001). A missing vas. *Journal of the Royal Society of Medicine*.

[4] Hautmann R., Huland H. (2006). *Urologie*.

[5] Shapiro E., Goldfarb D. A., Ritchey M. L. (2003). The congenital and acquired solitary kidney. *Reviews in Urology*.

[6] Carlson B. (2014). *Human Embryology and Developmental Biology*.

[7] Daudin M., Bieth E., Bujan L., Massat G., Pontonnier F., Mieusset R. (2000). Congenital bilateral absence of the vas deferens: clinical characteristics, biological parameters, cystic fibrosis transmembrane conductance regulator gene mutations, and implications for genetic counseling. *Fertility and Sterility*.

[8] Casals T., Bassas L., Egozcue S. (2000). Heterogeneity for mutations in the CFTR gene and clinical correlations in patients with congenital absence of the vas deferens. *Human Reproduction*.

[9] Schlegel P. N., Shin D., Goldstein M. (1996). Urogenital anomalies in men with congenital absence of the vas deferens. *Journal of Urology*.

